# Is Healthier Nutrition Behaviour Associated with Better Self-Reported Health and Less Health Complaints? Evidence from Turku, Finland

**DOI:** 10.3390/nu7105409

**Published:** 2015-10-14

**Authors:** Walid El Ansari, Sakari Suominen, Gabriele Berg-Beckhoff

**Affiliations:** 1 Faculty of Applied Sciences, University of Gloucestershire, Gloucester GL2-9HW, UK; 2 Department of Public Health, University of Turku, Turku FIN-20014, Finland; suominen@utu.fi; 3 Department of Public Health, University of Skövde, Skövde S-54128, Sweden; 4 Folkhälsan Research Center, FIN-00251 Helsinki, Finland; 5 Unit for Health Promotion Research, Institute of Public Health, University of Southern Denmark, 6700 Esbjerg, Denmark; gbergbeckhoff@health.sdu.dk

**Keywords:** Finland, food intake, health complaints, gender, student health, eating healthy, dietary guidelines adherence

## Abstract

We examined nutrition behaviour, self-reported health and 20 health complaints of undergraduates in Finland. Students at the University of Turku in Finland participated in a cross-sectional online survey (*N* = 1189). For nutrition behaviour, we computed two composite food intake pattern scores (sweets, cakes and snacks; and fruits and vegetables), a dietary guideline adherence index and the subjective importance of healthy eating. Multinomial logistic regression assessed the association of students’ nutrition behaviour with three levels of self-reported health, controlling for many potential confounders (age, sex, living with partner, economic situation, moderate physical activity, Faculty and BMI). Factor analysis of the 20 health complaints revealed three components (psychological, pains/aches and circulatory/breathing symptoms). Multiple linear regression tested the association of students’ eating habits with the three components of health complaints, controlling for the same confounders. Fruits and raw and cooked vegetable consumption, dietary guideline adherence index and subjective importance of healthy eating were highest among students with excellent/very good self-reported health, exhibiting a decreasing trend for those individuals with poor/fair self-reported health. High levels of psychological symptoms were associated with decreased consumption of fruits and vegetables, less dietary guideline adherence and less subjective importance of healthy eating. Pain/aches symptoms were associated with a higher consumption of sweets, cookies and snacks and a lower adherence to dietary guidelines. More healthy nutrition behaviour was consistently associated with better self-reported health and less health complaints. Of the four nutrition behaviour indicators we employed, the dietary guideline adherence index was the best indicator and exhibited the most consistent associations with self-reported health and health complaints.

## 1. Introduction

Healthy nutrition behaviour is an important component that is required to maintain health. However, there is only limited research examining this relationship, as many studies focused on the associations between nutrition behaviour/eating habits and disease (rather than health), e.g., [1,2,3]. As regards health, some research examined the associations between nutritional status and quality of life; a population-based cohort study found that adherence to a Mediterranean diet (MD) pattern was associated with better health-related quality of life and that the association was stronger with mental health than with physical health [4]. In addition, two systematic reviews on the associations between nutritional status and quality of life concluded that few studies were available that explored the relationships between nutrition and quality of life or health [5,6]. The scarcity of such research on the links between nutrition habits and health is further demonstrated by an older review [5], which found only three studies that reported a positive association between nutritional status and quality of life. Whereas the more recent review [6] was based on a larger sample of published papers (13 relevant studies), unfortunately, the authors only evaluated the tools (questionnaires) that were used to determine the association between dietary habits and quality of life, and therefore, no results on the links between nutrition behaviour and health were presented.

Such a paucity of research on the relationships between nutrition behaviour and health exists despite that the health behaviour patterns throughout life are largely established in the young adulthood period and that certain foods/nutrients can moderately influence several health outcomes. In France, compliance with nutrition guidelines was positively associated with mental health [7]. Similarly, in Chile, the importance of healthy eating was one of five factors that characterised the students according to their different eating habits and explained the greater satisfaction with life [8]. Students with higher levels of life satisfaction and satisfaction with food-related life reported fewer health problems, had healthful eating habits and considered food very important for their well-being [9].

In addition to the general scarcity of studies globally on the links between nutrition behaviour and health, very few studies on eating habits have been undertaken in Finland among university students and across a range of food groups in order to examine the multiple aspects of healthy eating behaviours, adherence to dietary guidelines, subjective importance of healthy eating and their two-pronged relationships to: (1) health features (e.g., self-reported health); and (2) unhealthy symptoms (e.g., health complaints). Some dietary research in Finland was population based [10], among adolescents [11] and elementary school pupils [12], or explored the intake of isolated food groups, e.g., daily vegetables [10] or bread [13]. Hence, Finnish studies on students’ nutrition behaviour and adherence to dietary guidelines are extremely lacking. The present study bridges these knowledge gaps, thus attaching high importance to the findings of the current research.

Several approaches are employed in order to estimate healthy nutrition behaviour/eating habits, particularly when examining many food groups rather than isolated individual foods. In a simple manner, the extent of consumption of the different kinds of healthy food groups (e.g., fruits and vegetables) has been used to estimate healthy nutrition behaviour [14,15,16]. Conversely, an alternative approach assessed healthy nutrition behaviour by the extent of omission (non-consumption) of the less healthy foods (e.g., sweets and snacks) [17,18,19]. In addition, the appraisal of the extent of compliance/adherence with general nutritional guidelines allows an overall view of healthy nutrition behaviour [7,15,18]. Likewise, studies [8] have shown that an individual’s own perception of the importance of healthy eating is critical with regards to a healthy life style; and research has similarly employed such subjective importance of healthy nutrition behaviour in examining the links between food and mood [15,18].

University students are the future of families, communities and countries and are an important population of young adults that face the challenges of, e.g., trying to achieve academic success despite financial constraints, and stressors that include personal expectations, peer competition, having to attain good grades or fear of failing/repeating their course. They are expected to be competitive, and the university period is one of responsibility for choices and lifestyle practices, where students are exposed to the challenges of young adulthood and also tackle the mental and social issues of student life. They confront changes in living conditions and (health promoting/damaging) adjustments to lifestyle and the environment, where a key concern is the food consumption patterns and associated nutritional risks specific to college students.

Given the knowledge gaps highlighted above, the aim of the current analysis is to assess the different aspects of healthy eating behaviours on health features (self-reported health) and unhealthy symptoms (health complaints). We assessed students’ two food intake pattern scores (health and unhealthy food groups), a dietary guidelines adherence index and the objective importance of healthy eating and their relationships with self-reported health and three groups of health complaints.

## 2. Methods

### 2.1. Sample, Ethics and Procedures

The research and ethics committee at the University of Turku in Turku, Finland, approved the study. Data were collected using a secure online survey in the English language (academic year 2013–2014). An invitation email with the research aims and objectives was sent in September 2013 to all (*n* = 4387) first, second and third year undergraduates at all faculties at the University, inviting students to participate by completing the online survey. We employed universal sampling, where all students were invited to participate in the study, with no inclusion/exclusion criteria. Participation was voluntary and anonymous, and data were confidential and protected. Students received contact information in case they had questions and were informed that by completing the survey, they agree to participate in the study. Two weeks later, a follow-up reminder email was sent again to the same undergraduate sample. Respondents completed the online survey and submitted their electronic responses that were automatically saved and sent to the Student Management Office at the University of Turku. The total number of responses was 1189 (response rate: 27.1%). For the current analysis, we excluded participants with missing data in nutrition intake and health complaints, reducing the number to 1027 students; 302 males (29.4%) and 725 females (70.6%). Participating students were enrolled at the seven faculties of the University: Humanity (31.3%), Mathematics and Natural Science (21.2%), Medicine (12.8%), Law (6.5%), Social Science (8.4%), Education (8.1%) and Economics (12.0%). The questionnaire collected general self-reported health data: socio-demographic information (gender, age, year of study, Faculty, living arrangements during university terms); lifestyle behaviours and a short food frequency questionnaire; the questionnaire has been used and field-tested across many student populations [20,21,22].

### 2.2. Questionnaire

Self-reported health was assessed with the question “How would you describe your general health” Responses were coded in a five-point scale from excellent to poor. For the analysis, three categories were built: (1) excellent and very good; (2) good; (3) fair and poor.

Assessment of self-reported health complaints (22 items): Students were asked how often they have had health complaints in the last year. Responses were coded in a four-point scale from never to very often. The following symptoms were asked about: depressive mode, nervousness/anxiety, mood swings, difficulties to concentrate, fear/phobia, sleep disorders/insomnia, nightmares, fatigue, lack of appetite, stomach trouble/heartburn, abdominal problems, neck and shoulder pain, back pain, diarrhoea, constipation, headaches, trembling hands, trembling, rapid heartbeat/circulatory problems, breathing difficulties, weight gain/weight loss and speech impediment. The last two symptoms were dropped from further analysis, because of unclear precision (weight gain/weight loss) and too many missing responses (e.g., item on speech impediment). Given the result from the factor analysis, three components were developed with nine variables for psychosomatic complaints (Cronbach’s alpha = 0.86), seven variables for pain and aches (Cronbach’s alpha = 0.73) and, finally, four variables for cardiovascular symptoms (Cronbach’s alpha = 0.74).

Assessment of food consumption habits (12 items): Students self-reported their nutritional behaviour in a food frequency questionnaire (FFQ) comprising 12 variables that measured their consumption of sweets, cakes/cookies, snacks and fast/canned food, fresh fruits, raw and cooked vegetables and salads, meat and fish, milk products and cereals. The introductory question, “How often do you eat the following foods?” queried students about the frequency of their usual consumption of each food group individually (5-point scale: “several times a day”, “daily”, “several times a week”, “1–4 times a month” and “never”). The question was asked generally to get information about the food consumption during a whole year. The instrument was based on pre-existing food frequency questionnaires, adapted for the study. Both the face and content validity of the instrument were ascertained by grounding the questionnaire on a literature review. No formal test of validity was performed, but the questionnaire was very similar to other food frequency questionnaires that had been validated, e.g., [23,24]. For descriptive purposes, the categories “several times a week” and “daily” were collapsed together. Then, in order to bring together (bundle) each of the healthy and less healthy food groups, two composite food intake pattern scores were developed (*a posteriori*-derived). The first was for the less healthy options, sweets, cake/cookies and snacks, where their relevant 5-point scales (see above) were added (summed up); and the second food intake pattern score was for the healthier options, fruits, raw and cooked vegetables, where their relevant 5-point scales were added (summed up).

The dietary guideline adherence index was computed using the FFQ. For sweets, cake/cookies, snacks, fast food/canned food and lemonade/soft drinks, no specific guidelines exist; hence, we employed “1–4 times a month” and “never”, as recommended. To consider all sweets, cake/cookies and snacks together, we used the above composite food intake pattern score (sweets, cookies and snacks score), and healthy eating was considered present if this score was ≤6 corresponding, to 3-times the intake of these items of “less often than 1–4 times a month”. Each of the fast food/canned food and lemonade/soft drinks were included as individual items in computing the objective guideline adherence index. For the remaining food groups, we used the WHO dietary guidelines recommendations for the European region [25]. Consequently, for the number of daily fruit, raw and cooked vegetable servings, the cut-off was “daily” or “several times a day”. For meat, the cut-off was “less than daily”; and for fish, “several times per week” was the cut-off. Milk and cereals were not included in the computing of the dietary guideline adherence index, as the information about milk and cereals was generally too unspecific in order to categorize as healthy or unhealthy nutrition. The dietary guideline adherence index has a maximum of 8 points (8 guidelines) calculated from 8 food group recommendations: (1) sweets, cookies, snacks; (2) fast food/canned food; (3) lemonade/soft drinks; (4) fruits; (5) salad, raw vegetables; (6) cooked vegetables; (7) meat; and (8) fish.

Importance of eating healthy (1 item): “How important is for you to eat healthy?” on a 5-point scale (1 = “Not at all important” to 5 = “very important”).

Socio-demographic and lifestyle variables (potential confounders): Due to their possible associations with eating behaviour, other variables employed in the analysis (potential confounders that were controlled for in the regression analysis) included: (1) age; (2) gender; (3) economic situation (“How sufficient is your income?”, coded into sufficient *vs.* not sufficient); (4) living situation/arrangements during university terms (“Where do you live during university term time?”, coded into living with partner *vs.* not living with partner); (5) moderate physical activity (PA) (“On how many of the past 7 days did you participate in moderate exercise for at least 30 minutes?”); participants answered with 0–7 days; we used a cut-off of ≥5 days/week as adherence to PA guidelines [26]; and, (6) BMI was calculated (kg/m^2^) and categorized into underweight (BMI < 18.5 kg/m^2^), normal weight (18.5–25 kg/m^2^) and overweight (>25 kg/m^2^) [27]. The overweight category also included the obese students (BMI > 30 kg/^2^). In addition, Faculty was also included as a potential confounder.

### 2.3. Statistical Analysis

Analysis was conducted in SAS Version 9.4 (SAS Institute, Cary, NC, USA) (*p* < 0.05). Factor analysis was employed in order to reduce the scale of health complaints to the main factors. Due to similar studies [17,22] and to facilitate the interpretation of the findings, a three-factor solution was selected with good factor loadings. The associations between food pattern and self-reported health were analysed using multinomial logistic regression, employing students who rated their health as excellent or very good as the reference group. Analyses were adjusted for age, sex, living situation, economic situation, moderate physical activity, faculty and BMI. The associations between food pattern and three different health complaints’ scores were analysed employing multiple linear regression models. Standardized beta-coefficients were additionally presented in order to allow the comparison of results between the three different health complaint scores. Data were also adjusted for age, sex, living situation, economic situation, moderate PA, faculty and BMI. Model assumptions were graphically tested and were fulfilled for the psychological complaint score and also for the pains/aches complaint score, but not for the circulatory/breathing complaint score (residuals were not normally distributed). Hence, the multiple linear regression analysis with the outcome of circulatory/breathing complaints results should therefore be interpreted with caution.

## 3. Results

Table 1 shows the socio-demographic characteristics and intake of 12 food groups for the whole sample and by gender. For both genders, the age ranged from 17–65 years (median = 21 years). Less than a third of students lived with their partners, and ≈40% felt they had sufficient money. Thirteen percent of women and 30% of men were overweight (BMI > 25 kg/m^2^). Both genders rarely ate sweets, cakes and cookies, did not commonly eat fast food and did not drink soft drinks on a daily basis. Both genders ate fruits and vegetables regularly (daily/several times a day).

**Table 1:** Socio-demographic and lifestyle characteristics and intake of 12 food groups by gender among undergraduates, University of Turku, Finland, 2013–2014.

	Males	Females	*p* **
	*N* (%)	*N* (%)	
	302 (29.4)	725 (70.6)	
**Age group** (years)			0.04
<20	78 (25.8)	232 (32.0)	
20–25	183 (60.6)	376 (51.9)	
≥25	41 (13.6)	116 (16.0)	
**Living situation**			0.02
Living with partner	71 (23.7)	220 (30.5)	
**Economic situation**			0.41
Always/mostly sufficient	120 (40.3)	309 (43.1)	
**Moderate physical activity**			0.64
Adherence to guideline	45 (15.0)	116 (16.1)	
**BMI** (reported)			<0.0001
Underweight (≤18.5 kg/m^2^)	10 (3.3)	49 (6.8)	
Normal (18.5–25 kg/m^2^)	201 (65.6)	581 (80.1)	
Overweight (>25 kg/m^2^)	91 (30.1)	95 (13.1)	
**Food frequency questionnaire (FFQ)** *			
Sweets	7 (2.3)	58 (8.0)	0.0007
Cake, cookies	1 (0.3)	11 (1.5)	0.11
Snacks	1 (0.3)	4 (0.5)	0.64
Fresh fruits	108 (35.8)	429 (59.1)	<0.0001
Salad, raw vegetables	173 (57.3)	525 (72.4)	<0.0001
Cooked vegetables	60 (19.9)	233 (32.1)	<0.0001
Fast food, canned food	3 (1.0)	1 (0.1)	<0.0001
Lemonade, soft drinks	19 (6.3)	21 (2.9)	0.01
Meat, sausages	165 (54.6)	215 (29.6)	<0.0001
Fish, sea food	19 (6.3)	15 (2.1)	0.0006
Milk, milk products	232 (76.8)	560 (77.2)	0.88
Cereals, cereal products	133 (44.0)	429 (59.2)	<0.0001
**Self-reported health**			0.001
Excellent/very good	178 (58.9)	330 (52.4)	
Good	94 (31.1)	303 (41.8)	
Fair/poor	30 (9.9)	42 (5.7)	
**Faculty**			<0.0001
Humanity	61 (20.3)	257 (35.7)	
Mathematics Natural Science	95 (31.5)	120 (16.7)	
Medicine	50 (16.6)	86 (12.0)	
Law	8 (2.7)	54 (7.5)	
Social Science	24 (7.8)	63 (8.8)	
Education	15 (5.0)	65 (9.0)	
Economics	48 (16.0)	74 (10.3)	

* Percentages calculated for intake of “several times per day” or “daily”; ** chi-square test.

Table 2 depicts the factor analysis of students’ health complaints into three components and their factor loadings. The first component included psychological symptoms, e.g., depressive mood, nervousness/anxiety, mood swings, difficulties to concentrate, fear/phobia, sleep disorders/insomnia, nightmares, fatigue and lack of appetite. The second component comprised pain and ache symptoms, e.g., stomach trouble/heartburn, abdominal problems, neck and shoulder pain, back pain, diarrhoea, constipation and headaches. Finally, the third component encompassed circulatory/breathing symptoms, e.g., trembling hands, trembling, rapid heartbeat/circulatory problems and breathing difficulties.

**Table 2:** Factor analysis of 20 health complaints into three components.

Health Complaint	Component		
	1 Psychological (9 items)	2 Pains/aches (7 items)	3 Circulatory/breathing (4 items)
Depressive mood	0.76		
Nervousness/anxiety	0.67		
Mood swings	0.66		
Difficulties to concentrate	0.57		
Fear/phobia	0.54		
Sleep disorders/insomnia	0.47		
Nightmares	0.44		
Fatigue	0.43		
Lack of appetite	0.41		
Stomach trouble/heartburn		0.62	
Abdominal problems		0.55	
Neck and shoulder pain		0.52	
Back pain		0.44	
Diarrhoea		0.44	
Constipation		0.42	
Headaches		0.38	
Trembling hands			0.74
Trembling			0.64
Rapid heartbeat/circulatory problems			0.51
Breathing difficulties			0.38

Note: Varimax rotation.

Table 3 depicts students’ health complaints, two food intake pattern scores, dietary guideline adherence index and the subjective importance of healthy eating by gender and by self-reported health categories. Women reported more health complaints than men and also ate more sweets, cookies, snacks and more fruits and vegetables than men. Additionally, women adhered more often to dietary guidelines and regarded healthy eating as important more than men. The three health complaint scores (psychological, pains/aches, and circulatory/breathing symptoms) were worse (*i.e.*, higher scores) for students who reported worse self-reported health, exhibiting a distinct and consistent stepladder appearance. Students’ consumption of sweets, cookies and snacks did not differ by the self-reported health categories. In contrast, fruits and raw and cooked vegetable consumption, dietary guideline adherence index, as well as the subjective importance of healthy eating were highest among students who reported excellent/very good self-reported health and exhibited in all three scores a decreasing trend (stepladder appearance) compared to those individuals with poor/fair self-reported health.

The association between healthy eating habits and self-reported health is shown in the multinomial logistic regression model adjusted for many potential confounders (Table 4). The sweets, cookies and snack score was not associated with self-reported health. High fruit and vegetables consumption, good dietary guideline adherence and subjective importance of health eating were all less strongly associated with poor/fair self-reported health and increased in a linear manner with better self-reported health. This trend was most pronounced with the subjective importance of healthy eating, followed by fruits and vegetable consumption. The trend was less pronounced with the dietary guideline adherence.

**Table 3:** Health complaints and eating habits of undergraduates by gender and self-reported health, University of Turku, Finland, 2013–2014.

	Gender		Self-Reported Health		
	Male *N* = 302	Female *N* = 725	Excellent/Very Good *N* = 558	Good *N* = 397	Fair/Poor *N* = 72
	M(SD)	M(SD)	M(SD)	M(SD)	M(SD)
**Health complaints**					
Psychological symptoms score *^1^	16.39(5.18)	19.74(5.28)	16.77(4.57)	20.43(5.26)	24.82(5.50)
Pains/aches symptoms score *^2^	12.86(3.03)	15.81(3.52)	13.73(3.34)	16.21(3.54)	17.36(3.82)
Circulatory/breathing symptoms score *^3^	5.58(1.97)	6.12(2.22)	5.34(1.68)	6.45(2.26)	7.96(2.91)
**Food intake pattern score**					
Sweets, cookies, and snacks **	6.32(1.27)	6.66(1.17)	6.52(1.21)	6.64(1.21)	6.44(1.19)
Fruit, and raw and cooked vegetable **	9.49(1.93)	10.74(1.93)	10.61(1.97)	10.21(1.91)	9.42(2.45)
**Dietary guideline adherence index ^†^**	4.03(1.68)	4.93(1.63)	4.81(1.68)	4.59(1.66)	3.94(1.86)
**Subjective importance of healthy eating ^††^**	3.78(0.92)	4.10(0.77)	4.16(0.78)	3.88(0.82)	3.56(0.98)

M: mean; SD: standard deviation; *^1^ range: 4–36, higher values correspond to more perceived psychological symptoms; *^2^ range: 4–28, higher values correspond to more perceived pains/aches symptoms; *^3^ range: 4–16, higher values correspond to more perceived cardiovascular/breathing symptoms; ** range: 3–15, each score increases as more is eaten; ^†^ range: 1–8, each point increase represents an additional food group that shows adherence to dietary guidelines; ^††^ range: 1–5, higher values indicate higher importance.

**Table 4:** Multinomial logistic regression of nutrition behaviours on self-reported health among undergraduates by gender, University of Turku, Finland, 2013–2014.

	Self-Reported Health		
	Excellent/Very Good	Good	Fair/Poor
	OR	OR (95% CI)	OR (95% CI)
Food intake pattern score			
Sweets, cookies, and snacks	1 (reference)	1.03 (0.92–1.15)	0.91 (0.73–1.13)
Fruits, and raw and cooked vegetables	1 (reference)	**0.92 (0.85–0.99)**	**0.80 (0.70–0.92)**
Dietary guideline adherence index	1 (reference)	0.94 (0.86–1.01)	**0.82 (0.70–0.96)**
Subjective importance of healthy eating	1 (reference)	**0.69 (0.57–0.82)**	**0.51 (0.38–0.69)**

OR: odds ratio; CI: confidence interval; models adjusted for age, sex, living with partner, economic situation, moderate physical activity, faculty and BMI; bolded cells indicate statistical significance (*p* < 0.05).

Table 5 shows the association between healthy eating habits and health complaints. In this multiple linear regression model (adjusted for age, sex, living with partner, economic situation, moderate physical activity, Faculty and BMI), high levels of psychological symptoms were associated with decreased consumption of fruits and vegetables, less dietary guideline adherence and less subjective importance of healthy eating. Pain/aches symptoms were associated with a higher consumption of sweets, cookies and snacks and a lower adherence to dietary guidelines. Finally, circulatory/breathing symptoms were not explained well by this multiple linear regression model on eating habits, as the model fit was poor; despite this, we observed that students with circulatory/breathing symptoms less often viewed healthy eating as important and also adhered to dietary guidelines less often.

Table 6 summarises the utility of employing such different nutritional behaviour (variables) indicators for health (different levels of self-reported health) and “unhealthy” (*i.e.*, different health complaints).

**Table 5:** Multiple linear regression of nutrition behaviours on health complaints among undergraduates by gender, University of Turku, Finland, 2013–2014.

	Health Complaints					
	Psychological		Pains/Aches		Circulatory/Breathing	
	Std-ß	β (95% CI)	Std-ß	β (95% CI)	Std-ß	β (95% CI)
Food intake pattern score						
Sweets, cookies, snacks	0.02	0.08 (−0.18; 0.34)	**0.10**	**0.30 (0.12; 0.47)**	0.03	0.05 (−0.06; 0.16)
Fruits, raw and cooked vegetables	−0.05	−0.13 (−0.30; 0.04)	0.01	0.01 (−0.09; 0.13)	−0.04	−0.04 (−0.10; 0.03)
Dietary guideline adherence index	**−0.06**	**−0.20 (−0.40; −0.01)**	**−0.09**	**−0.20 (−0.33; −0.06)**	−0.03	−0.04 (−0.12; 0.04)
Subjective importance of healthy eating	**−0.08**	**−0.50 (−0.89; −0.11)**	**−0.01**	−0.04 (−0.30; 0.23)	**−0.07**	**−0.19 (−0.35; −0.02)**

Std-ß: standardized beta coefficient; ß: beta coefficient; CI: confidence interval; models adjusted for age, sex, living with partner, economic situation, moderate physical activity, faculty and BMI; bolded cells indicate statistical significance (*p* < 0.05).

**Table 6:** Utility of different nutritional behaviour indicators for different levels of self-reported health and different health complaints.

Nutritional Behaviour Indicator Used	Outcome Used				
	Self-Reported Health		Health Complaints		
	Good *	Fair/Poor *	Psychological	Pains/Aches	Circulatory/Breathing
Food intake pattern score					
Sweets, cookies and snacks	No	No	No	Yes	No
Fruits, and raw and cooked vegetables	Yes	Yes	No	No	No
Dietary guideline adherence index	No	Yes	Yes	Yes	No
Subjective importance of healthy eating	Yes	Yes	Yes	No	Yes

* As compared to “excellent/very good” self-reported health.

## 4. Discussion

We observed that less healthy nutrition behaviour was consistently associated with worse self-reported health and more health complaints. Of the four nutrition behaviour indicators we employed, the dietary guideline adherence index revealed the most consistent association with self-reported health and more health complaints. For a better understanding, self-reported health and health complaints are discussed separately.

### 4.1. Nutritional Behaviour Indicators and Self-Reported Health

For the aspects of self-reported health, we observed that two nutrition behaviour indicators (fruits, and raw and cooked vegetables intake score and subjective importance of healthy eating) were more sensitive to various levels of self-reported health than the dietary guideline adherence index, a point to note for nutrition intervention programs in selecting appropriate indicators in order to track their success. In addition to the above, we observed that the sweets, cookies and snacks score (unhealthy food score) was not associated with self-reported health. This finding suggested: (1) such an unhealthy food score might not be suitable for nutrition campaigns that tackle the composition of unhealthy foods and simultaneously use self-reported health as a measure to trace their own progress; and (2) individuals seem not to view sweets, cookie and snack consumption as having much to do with their health, a very erroneous point that needs to be addressed by health nutrition intervention initiatives.

Despite the difficulty of locating published studies that are directly comparable to the current research, our findings that, generally, better nutritional behaviour was associated with better (excellent/very good) self-reported health agree with parallel literature. At the population level, in Spain, adherence to the Mediterranean diet (MD) was associated with a higher self-perceived health score [28]; and across university students in 21 countries, a healthful diet was positively related to greater life satisfaction [29]. Likewise, we found that only the consumption of fruits and raw and cooked vegetables (as opposed to sweets, cookies and snacks) were associated with better (excellent/very good) self-reported health, in support of the fact that life satisfaction was positively associated with eating fruit in students aged 17–30 [29]; and in agreement with university students in Chile, where only fruit consumption was linked to a higher satisfaction with life [9]. Similarly, on the population level, in Italy, adherence to an MD pattern (primarily plant-based foods, e.g., fruits and vegetables) was associated with better health-related quality of life, which is the subjective evaluation of one’s own health (i.e., self-reported health) and well-being [4,30].

### 4.2. Nutritional Behaviour Indicators and Health Complaints

In terms of the different health complaints, we observed selectivity in the associations between nutrition behaviour (different food groups) and different health complaints. Hence, the consumption of more fruits and raw and cooked vegetables was more associated with less psychological health complaints; whereas conversely, regularly eating sweets, cookies and snacks was more associated with more pains and aches symptoms. These selectivity findings suggested direct implications for prevention programs among university student populations. Strategies and policies aimed at promoting healthy nutrition behaviours (food) are likely to simultaneously have beneficial effects on mental health (mood). Likewise, regularly eating fruits and raw and cooked vegetables was associated with better psychological health. Because of the cross-sectional design of the current study, we are unable to make solid conclusions about the direction of any of the associations we observed. The relationships could also be bi-directional; whether worse psychological health influences eating less fruits and vegetables or more pain and aches influences consuming more sweets, snacks and cookies; or conversely, does nutrition behaviour influence health complains? In our opinion, it might be more plausible that nutrition behaviour is influenced by health complaints. Further research would help with understanding these relationships.

Our finding that consumption of more fruits and raw and cooked vegetables was more associated with less psychological health complaints supports other research. At the level of adolescents, in Greece, good adherence to the MD (better diet quality) was associated with better psychological health [31]; and for university graduates, in Spain, multivariate-adjusted models revealed a significant direct association between MD adherence (high in fruits/vegetables) and most mental health domains (vitality, social functioning and role emotional) [32]. Our finding also agrees with the inverse relationship between fruits and vegetable consumption and psychological health (e.g., perceived stress and depressive symptoms) demonstrated across university students of both genders in the United Kingdom [17], among female undergraduates in Germany, Poland and Bulgaria [33] and among college students in China [34].

Another feature of selectivity in terms of the relationships between nutrition behaviour and health complaints) was between the different health complaints and the various nutrition behaviour indicators. Psychological health complaints were more consistently associated with three of the four nutrition behaviour indicators; as opposed to the pains/aches complaints and the circulatory/breathing complaints, where each was associated with only two of the nutrition behaviour indicators we used. Hence mood (psychological symptoms) might be more sensitive to healthy nutrition behaviour indicators than other health complaints and would be better employed as an outcome measure to calibrate the effectiveness of prevention polices and nutritional (food) campaigns that foster healthful eating in university populations. Future research would assist in uncovering the details of these associations.

A further aspect of selectivity in terms of the aspects of health complaints was between the various indicators we employed and the different health complaints. Of the four indicators that were employed, the dietary guideline adherence index exhibited the most sensitivity to health complaints (associated with all three different groups of health complaints that were examined). This was followed by the subjective importance of healthy eating indicator (associated with only two of the three groups of health complaints). The least sensitive indicators were the food intake pattern scores, where each was only associated with only one of the three groups of health complaints. Such findings suggested that: (1) dietary guideline adherence indices or similar measures would be more preferable over other nutrition behaviour indicators when examining the relationships between food and health complaints/symptoms; (2) using individual food groups in isolation (e.g., either “healthy” and “unhealthy” food intake pattern scores) might be unhelpful when examining the relationships between food and health complaints; and, (3) for prevention strategies/intervention programs that target students’ psychosomatic complaints and self-reported symptoms, highlighting the importance of adhering to nutritional guidelines and recommendations should be an important component of such efforts. Future research would assist in unearthing more features of these associations.

The paucity of research of such relationships rendered it challenging to compare our findings to other published work. However, our finding of the sensitivity of the dietary guideline adherence index supports some of the studies across the globe that used adherence levels to a particular diet (e.g., MD) [4,7,27,31], compliance with international nutritional guidelines [15,18] or the adequacy in following national nutritional recommendations [35] when examining the relationships between nutrition behaviour and a variety of outcomes. Nevertheless, whilst most of these studies used only an adherence index (perhaps blindly or for convenience), the current study undertook the arduous task of employing four different nutritional behaviour indicators to explore their utility in research and practice, as highlighted in Table 6.

This study has some limitations. We undertook the study at one university in Finland, and the response rate was not very high; hence, generalizations need to be cautious. Self-reports are prone to sociability/social desirability with the implication that the current findings could represent an underestimation of less healthy nutritional habits (e.g., sweets, cakes and cookies); no objective food consumption was undertaken; self-reporting was used to estimate the frequency of symptoms, and clinical validations were not undertaken. For some food groups, we did not assess serving sizes. As this survey was cross-sectional, the direction of the association (temporal relationship) between food intake and health complaints cannot be ascertained. Students with higher health complaints, worse self-reported health, or those with more unhealthy patterns of eating might have chosen not to participate in the study, and selection bias cannot be ruled out. Therefore, the reported prevalence of symptoms in the current study may under-estimate the true morbidity in this population. It would have also been useful to assess whether any systematic differences existed between students who participated in the survey and those who did, but we were unable to undertake this task, as we had no data about those who did not participate in the survey. We did not control for participants’ co-morbidities that could affect subjects’ health practices and dietary behaviour. However, university populations traditionally represent young and healthy adults with minimal comorbidities. Future research should consider such limitations. Despite these limitations, the current research also has strengths. For data collection, our sample comprised students across seven faculties and many scientific disciplines. The sample comprised more females than males (a reality at higher education institutions across the globe), which could be associated with an increased likelihood of social desirability; hence, we analysed the relationships controlling for gender and other confounders to avoid potential confounding gender effects. For the analysis, we used WHO dietary guidelines that are appropriate for Finland. Recommendations for Nordic countries, as well as the recent Baltic Sea diet do exist [36,37], but they differ from WHO recommendations only in terms of food items within food groups (e.g., fruit and vegetables found in Nordic countries are different than the ones recommended by the Mediterranean diet), but not in terms of portions and frequency. There were very few missing values in the students’ responses (most students answered all of the food frequency questions), thus avoiding any potential effects of missing values on the observed adherence estimates and associations. No previous studies in Finland of university students undertook such tasks of the links between eating habits, diet quality and dietary guidelines adherence and health complaints.

## 5. Conclusions

Despite the scarce research available, in the current study, healthier nutrition behaviour was consistently associated with better self-reported health and less health complaints. Of the four nutrition behaviour indicators we employed, the dietary guideline adherence index was the best indicator and exhibited the most consistent associations with self-reported health and more health complaints. Prevention and intervention efforts that aim at improving awareness and compliance with dietary guidelines might also be associated with decreased health complaints and improved health of university students.

## References

[1] Guasch-Ferré M., Hruby A., Salas-Salvadó J., Martínez-González M.A., Sun Q., Willett W.C., Hu F.B. (2015). Olive oil consumption and risk of type 2 diabetes in US women. Am. J. Clin. Nutr..

[2] Aljefree N., Ahmed F. (2015). Association between dietary pattern and risk of cardiovascular disease among adults in the Middle East and North Africa region: A systematic review. Food Nutr. Res..

[3] Lachance L., Ramsey D. (2015). Food, mood, and brain health: Implications for the modern clinician. Mo Med..

[4] Bonaccio M., di Castelnuovo A., Bonanni A., Costanzo S., de Lucia F., Pounis G., Zito F., Donati M.B., de Gaetano G., Iacoviello L. (2013). Adherence to a Mediterranean diet is associated with a better health-related quality of life: A possible role of high dietary antioxidant content. BMJ Open.

[5] Wanden-Berghe C., Sanz-Valero J., Escribà-Agüir V., Castelló-Botia I., Guardiola-Wanden-Berghe R. (2009). Evaluation of quality of life related to nutritional status. Br. J. Nutr..

[6] Ruano-Rodríguez C., Serra-Majem L., Dubois D. (2015). Assessing the impact of dietary habits on health-related quality of life requires contextual measurement tools. Front Pharmacol..

[7] Germain L., Latarche C., Kesse-Guyot E., Galan P., Hercberg S., Briançon S. (2013). Does compliance with nutrition guidelines lead to healthy aging? A quality-of-life approach. J. Acad. Nutr. Diet..

[8] Schnettler B., Denegri M., Miranda H., Sepúlveda J., Orellana L., Paiva G., Grunert K.G. (2013). Eating habits and subjective well-being among university students in southern Chile. Nutr. Hosp..

[9] Schnettler B., Miranda H., Lobos G., Orellana L., Sepúlveda J., Denegri M., Etchebarne S., Mora M., Grunert K.G. (2015). Eating habits and subjective well-being. A typology of students in Chilean state universities. Appetite.

[10] Roos E., Talala K., Laaksonen M., Helakorpi S., Rahkonen O., Uutela A., Prättälä R. (2008). Trends of socioeconomic differences in daily vegetable consumption, 1979–2002. Eur. J. Clin. Nutr..

[11] Pohjanheimo T., Luomala H., Tahvonen R. (2010). Finnish adolescents’ attitudes towards wholegrain bread and healthiness. J. Sci. Food Agric..

[12] Laitinen P., Nissinen A., Myllykangas M. (1993). Fat consumption of first-grade students in elementary school. Quality and quantity of fats used at home and at school. Hoitotiede.

[13] Prättälä R., Helasoja V., Mykkänen H. (2001). The consumption of rye bread and white bread as dimensions of health lifestyles in Finland. Public Health Nutr..

[14] El Ansari W., Clausen S.V., Mabhala A., Stock C. (2010). How do I look? Body image perceptions among university students from England and Denmark. Int. J. Environ. Res. Public Health.

[15] El Ansari W., Suominen S., Berg-Beckhoff G. (2015). Mood and Food at the University of Turku in Finland: Nutritional correlates of perceived stress are most pronounced among overweight students. Int. J. Public Health.

[16] El Ansari W. (2014). Health and well-being of students at higher education institutions—Time for urgent action?. Cent. Eur. J. Public Health.

[17] El Ansari W., Adetunji H., Oskrochi R. (2014). Food and mental health: Relationship between food and perceived stress and depressive symptoms among university students in the United Kingdom. Cent. Eur. J. Public Health.

[18] El Ansari W., Suominen S., Samara A. (2015). Eating Habits and Dietary Intake: Is Adherence to Dietary Guidelines Associated with Importance of Healthy Eating among Undergraduate University Students in Finland?. Cent. Eur. J. Public Health.

[19] El Ansari W., Dibba E., Stock C. (2014). Body image concerns: Levels, correlates and gender differences among students in the United Kingdom. Cent. Eur. J. Public Health.

[20] El Ansari W., Stock C. (2010). Is the health and wellbeing of university students associated with their academic performance? Cross sectional findings from the United Kingdom. Int. J. Environ. Res. Public Health.

[21] El Ansari W., Stock C., Mikolajczyk R.T. (2012). Relationships between food consumption and living arrangements among university students in four European countries-a cross-sectional study. Nutr. J..

[22] El Ansari W., Oskrochi R., Haghgoo G. (2014). Are students’ symptoms and health complaints associated with perceived stress at university? Perspectives from the United Kingdom and Egypt. Int. J. Environ. Res. Public Health.

[23] Osler M., Heitmann B.L. (1996). The validity of a short food frequency questionnaire and its ability to measure changes in food intake: A longitudinal study. Int. J. Epidemiol..

[24] Roddam A.W., Spencer E., Banks E., Beral V., Reeves G., Appleby P., Barnes I., Whiteman D.C., Key T.J. (2005). Reproducibility of a short semi- quantitative food group questionnaire and its performance in estimating nutrient intake compared with a 7-day diet diary in the Million Women Study. Public Health Nutr..

[25] WHO (2003). Food Based Dietary Guidelines in the WHO European Region.

[26] Haskell W.L., Lee I.M., Pate R.R., Powell K.E., Blair S.N., Franklin B.A., Macera C.A., Heath G.W., Thompson P.D., Bauman A. (2007). Physical activity and public health: Updated recommendation for adults from the American College of Sports Medicine and the American Heart Association. Circulation.

[27] WHO (2000). Obesity: Preventing and Managing the Global Epidemic.

[28] Muñoz M.A., Fíto M., Marrugat J., Covas M.I., Schröder H., REGICOR and HERMES investigators (2009). Adherence to the Mediterranean diet is associated with better mental and physical health. Br. J. Nutr..

[29] Grant N., Wardle J., Steptoe A. (2009). The relationship between life satisfaction and health behavior. A cross-cultural analysis of young adults. Int. J. Behav. Med..

[30] Baumann C., Erpelding M.L., Perret-Guillaume C., Gautier A., Régat S., Collin J.F., Guillemin F., Briançon S. (2011). Health-related quality of life in French adolescents and adults: Norms for the DUKE Health Profile. BMC Public Health.

[31] Costarelli V., Koretsi E., Georgitsogianni E. (2013). Health-related quality of life of Greek adolescents: The role of the Mediterranean diet. Qual. Life Res..

[32] Henríquez Sánchez P., Ruano C., de Irala J., Ruiz-Canela M., Martínez-González M.A., Sánchez-Villegas A. (2012). Adherence to the Mediterranean diet and quality of life in the SUN Project. Eur. J. Clin. Nutr..

[33] Mikolajczyk R.T., el Ansari W., Maxwell A.E. (2009). Food consumption frequency and perceived stress and depressive symptoms among students in three European countries. Nutr. J..

[34] Liu C., Xie B., Chou C.P., Koprowski C., Zhou D., Palmer P., Sun P., Guo Q., Duan L., Sun X. (2007). Perceived stress, depression and food consumption frequency in the college students of China Seven Cities. Physiol. Behav..

[35] Estaquio C., Kesse-Guyot E., Deschamps V., Bertrais S., Dauchet L., Galan P., Hercberg S., Castetbon K. (2009). Adherence to the French Programme National Nutrition Sante Guideline Score is associated with better nutrient intake and nutritional status. J. Am. Diet. Assoc..

[36] Kanerva N., Kaartinen N.E., Schwab U., Lahti-Koski M., Mannisto S. (2013). Adherence to the Balti Sea diet consumed in the Nordic countries is associated with lower abdominal obesity. Br. J. Nutr..

[37] Uusitupa M., Hermansen K., Savolainen M.J., Schwab U., Kolehmainen M., Brader L., Mortensen L.S., Cloetens L., Johansson-Persson A., Onning G. (2013). Effects of an isocaloric healthy Nordic diet on insulin sensitivity, lipid profile and inflammation markers in metabolic syndrome—a randomized study (SYSDIET). J. Intern. Med..

